# A rare and complex presentation of congenital anomalies: meningomyelocele, corpus callosum agenesis, and dextrocardia in a neonate

**DOI:** 10.11604/pamj.2025.52.136.46847

**Published:** 2025-12-03

**Authors:** Sakshi Desai, Sharath Hullumani

**Affiliations:** 1 Department of Paediatrics Physiotherapy, Ravi Nair Physiotherapy College, Datta Meghe Institute of Higher Education and Research (DU), Sawangi (Meghe), Wardha, India

**Keywords:** Spina bifida, brain malformation, agenesis of the corpus callosum

## Image in medicine

**Figure 1:**
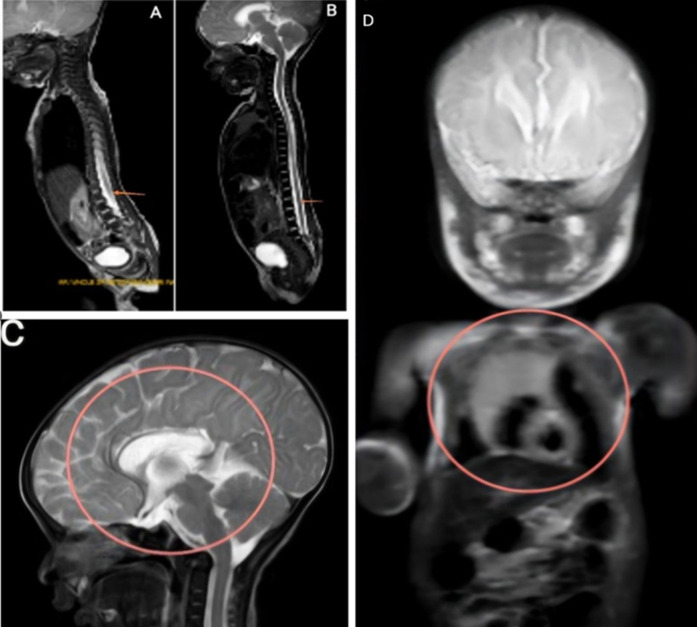
A) preoperative MRI of the whole spine showing a defect in the posterior elements of the sacral superior endplate; B) postoperative MRI of the whole spine showing hypointensity in the cord; C) brain MRI showing severe thinning of the genu and body of the corpus callosum; D) MRI of the whole body showing dextrocardia without situs inversus

A 2.2kg male neonate, born at 41 weeks of gestation via lower segment cesarean section (LSCS) to a gravida 4, para 3, live 3 (G4, P3, L3) mother. The neonate was transferred to the neonatal intensive care unit (NICU) due to the presence of soft tissue swelling on the lower back and meconium-stained liquor (MSL). Magnetic resonance imaging (MRI) of the whole spine was performed, which revealed spinal dysraphism in the lumbosacral region. Panel A shows a defect in the posterior elements of the sacral superior endplate of the S2 vertebra to the inferior endplate of the S4 vertebra. A neural placode with cerebrospinal fluid (CSF) was observed herniating into a saccular outpouching, measuring approximately 1.3 x 4.0 x 1.5 mm through a defect size of 5.2 x 13.3mm. Subcutaneous fat was seen covering the myelomeningocele sac. The nerve root appears thickened and isotense on T2- low-lying conus. The imaging features are suggestive of an open type of sacral meningomyelocele. Figure 1 (B) There is e/o T2WI hypointensity in the cord at the level of the lower endplate of L5 to the upper endplate of S2 vertebra. Post-operative changes are noted in the lower back. Panel C represents a brain MRI, which revealed severe thinning of the genu and body of the corpus callosum with agenesis of the splenium of the corpus callosum. Prominent bilateral lateral and third ventricles. Cavum septum pellucidum and cavum veli interpositi noted (anatomical variant). Panel D depicts the MRI whole body, which revealed dextrocardia without situs inversus. The three differential diagnoses included meningomyelocele, congenital anomalies, and spina bifida. The final diagnosis was callosum dysgenesis and dextrocardia. The neonate was managed in the NICU with a sterile dressing of the lesion, prone positioning, infection prevention measures, fluid/electrolyte monitoring, and oral motor stimulation.

